# Characterization of Non-Derivatized Cellulose Samples by Size Exclusion Chromatography in Tetrabutylammonium Fluoride/Dimethylsulfoxide (TBAF/DMSO)

**DOI:** 10.3390/molecules22111985

**Published:** 2017-11-16

**Authors:** Jérémy Rebière, Antoine Rouilly, Vanessa Durrieu, Frédéric Violleau

**Affiliations:** 1 Laboratoire de Chimie Agro-industrielle (LCA), Université de Toulouse, INRA, INPT, 31030 Toulouse, France; jeremy.rebiere@ensiacet.fr (J.R.); antoine.rouilly@ensiacet.fr (A.R.); 2 Laboratoire de Chimie Agro-industrielle (LCA), Université de Toulouse, INRA, INPT, INP-EI PURPAN, 31062 Toulouse, France; frederic.violleau@purpan.fr

**Keywords:** cellulose, molar mass, SEC analysis, conventional calibration

## Abstract

This paper deals with the use of tetrabutylammonium fluoride/dimethylsulfoxide (TBAF/DMSO) to characterize the molar mass distribution of non-derivatized cellulosic samples by size exclusion chromatography (SEC). Different cellulose samples with various average degree of polymerization (DP) were first solubilized in this solvent system, with increasing TBAF rates, and then analyzed by SEC coupled to a refractive index detector (RID), using DMSO as mobile phase. The Molar Masses (MM) obtained by conventional calibration were then discussed and compared with suppliers’ data and MM determined by viscosimetry measurements. By this non-classic method, molar mass of low DP samples (Avicel^®^ and cotton fibers) have been determined. For high DP samples (α-cellulose and Vitacel^®^), dissolution with TBAF concentration of 10 mg/mL involved elution of cellulose aggregates in the exclusion volume, related to an incomplete dissolution or the dilution of TBAF molecules in elution solvent, preventing the correct evaluation of their molar mass.

## 1. Introduction

Cellulose is a natural, biodegradable and biocompatible resource, and represents about 50% of the associated carbon on earth. Its interest as a source of fibers in paper industry, textiles and materials, and more recently as a source of glucose for bio-ethanol production or a novel sugar-based chemistry, is constantly growing. For all these applications, the determination of molar masses, both average value and distribution is more and more expected, as they are key parameters to control and improve the industrial processes as well as the final product properties.

Size Exclusion Chromatography (SEC) is nowadays the favorite technique for this type of characterization, and several analytical methods have been established since the early 70’s. They mainly differ from each other with respect to the cellulose dissolution step prior to SEC analysis. During the 70’s and 80’s, indirect dissolution was preferred, through the synthesis of non-polar cellulose derivatives, such as cellulose tricarbanilates (CTC) [1,2,3,4,5]. From the 90’s, direct dissolution methods, without any derivatization, using polar solvent mixtures like lithium chloride/*N*,*N*-dimethylacetamide (LiCl/DMAc) have emerged [6,7,8]. McCormick et al. [6] have used lithium chloride/*N*,*N*-dimethylacetamide (LiCl/DMAc) as solvent system for cellulose characterization by SEC, and reported the stability, solubility and dissolving states of cellulose in this solvent system. Subsequently, LiCl/DMAc has been favorably used for cellulose molar mass distribution (MMD) characterization particularly on birch wood Kraft pulp, hardwood or softwood Kraft pulps or aged papers [9,10,11,12,13,14,15]. Several works [16,17,18] reported cellulose dissolution in LiCl/DMAc without degradation, despite a slight decrease of the viscosity of cellulose solutions after 30 days has been observed [6]. However, some relevant problems concerning the use of LiCl/DMAc have been described: (i) the aggregation of cellulose in the solution depending on dissolution conditions and concentrations of cellulose or LiCl [19]; (ii) incomplete dissolution of several cellulose samples [20,21] and (iii) detrimental degradation of cellulose by heating in DMAc or LiCl/DMAc during the dissolution process [22], especially for high molecular weight samples. Consequently, cellulose characterization by SEC analyses using LiCl/DMAc as solvent is still currently a topic a numerous researches [23]. Other solvent systems have also been studied, such as LiCl/DMI described by Yanagisawa and Isogai [24,25].

TBAF/DMSO has been reported to be a good solvent for various cellulose samples, without affecting significantly their physical structure [26,27]. Even if the dissolution mechanism was not clearly explicated yet, it has been accepted that: (i) DMSO is an excellent swelling agent for cellulose, increasing the accessibility to hydrogen bonds; (ii) the fluoride ion, due to its strong hydrogen bond acceptor character, can then reduce the intermolecular attraction between the polysaccharide chains and disrupt the hydrogen bonds network [27]. For this reason, this system appears as a suitable candidate for SEC analysis of cellulose samples. Additionally, the replacement of a carcinogenic solvent such as DMAc by DMSO could be of interest for the health of the operators and for the environment. DMSO is classified as nontoxic with no risk for the human health according to US Environmental Protection Agency (EPA). Finally, DMSO is present in some industrial wastewaters and is readily biodegradable [28], and could be synthesized from renewable raw materials in the future.

The size exclusion separation mechanism is based on differences in the solute hydrodynamic volumes. Different methods can be used to obtain the molar mass of the components of chromatographed samples [29]. However, the most common and simplest method to define the relationship between the molar mass and elution volume is to calibrate the columns with narrowly distributed polymeric standards, using a refractive index detector. Pullulan standards are frequently used to obtain the MMD of cellulosic samples. Indeed, due to their linearity and the chemical structure (polymaltotriose units linked together by α-(1,6)-linkages), it is considered that the relation between molar mass and hydrodynamic volume is close to what is expected for cellulose [15,30,31,32].

In this study, TBAF/DMSO solvent was tested as a new greener solvent system for SEC analysis of four cellulose samples having various average degrees of polymerization (DP): Avicel^®^ and cotton fibers known to have low DP (<300), and α-cellulose and Vitacel^®^ chosen for their high DP (>1000). SEC was coupled to a refractive index detector and DMSO was used as mobile phase. The results obtained from pullulans calibration were then compared with suppliers’ data and viscosimetric DP measurements.

## 2. Results and Discussion

A preliminary study in which TBAF/DMSO was directly used as elution solvent was performed with various TBAF concentrations (2.5, 5 and 10 mg/mL). However, the column pressure limit (30 bars) was rapidly reached, indicating column saturation. The higher the TBAF concentration was, the faster the saturation occurred. TBAF molecules seem to interact with PS-DVB co-polymer composing the stationary phase of the column. Consequently, DMSO alone was used as elution solvent and cellulose and pullulan samples were dissolved in TBAF/DMSO solutions, before SEC analysis. Detection was performed only with a refractive index detector using pullulans calibration.

### 2.1. Influence of TBAF Concentration on Pullulan Calibration Curves

**Figure 1:**
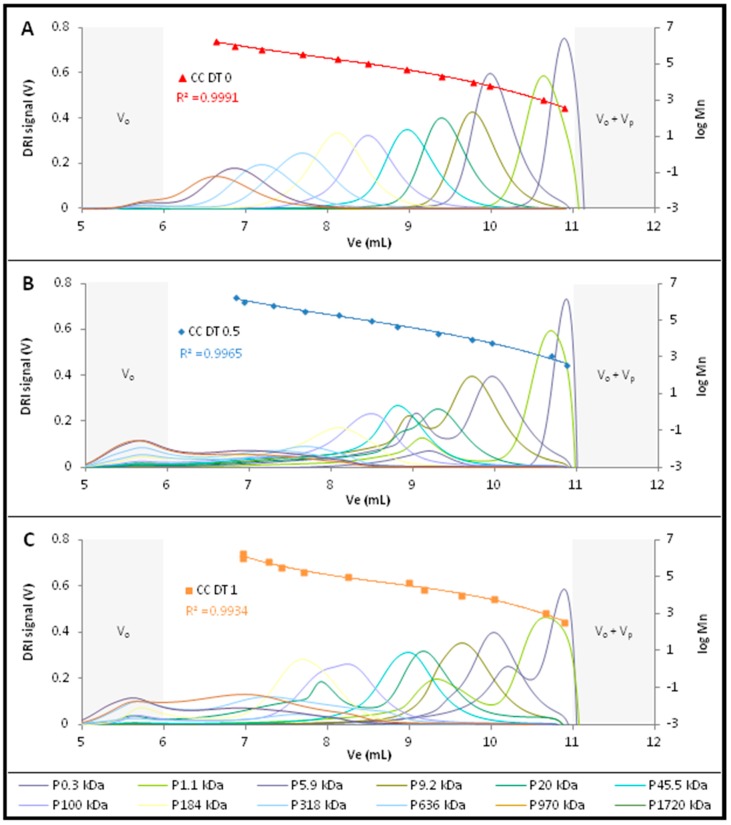
SEC calibration curves in DMSO (60 °C and 0.5 mL/min) of pullulan standards with different concentrations of TBAF: (**A**) 0 mg/mL; (**B**) 5 mg/mL; (**C**) 10 mg/mL.

Firstly, the influence of the TBAF concentration was studied on the pullulan standards. Three calibration curves with various TBAF concentrations 0, 5 and 10 mg/mL (named respectively CC DT 0, CC DT 0.5 and CC DT 1.0) were obtained (Figure 1) by polynomial regression, with satisfying correlation coefficients (R^2^ > 0.99%).

**Figure 2:**
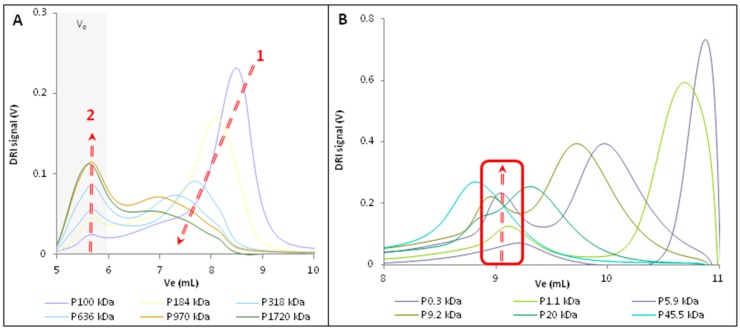
Increase of hydrodynamic volumes of pullulan standards prepared with 5 mg/mL of TBAF. (**A**) High molecular weight samples; (**B**) low molecular weight samples.

However, increasing TBAF concentration involved significant modifications of the elution peaks shape and position (Figure 2). In the case of the low molecular weight samples (Mn lower than 100 kDa), for each standard a new elution peak appeared in addition to the one observed without TBAF. This new peak had a lower elution volume, suggesting the apparition of a second population with a higher hydrodynamic volume in the sample (Figure 2B). The increase of the hydrodynamic volume with TBAF concentration in solution can be explained by the formation of TBAF aggregates on pullulan chains in the presence of TBAF/DMSO due to the formation of the complex {DMSO-TBAF-pullulan}.

For high molecular weight pullulan samples (higher than 100 kDa), this aggregation phenomenon had a slightly different consequence. The elution peaks were flattened, widened and displaced to lower elution volume, and an exclusion peak (elution volume between 5 mL and 6 mL) appeared and increased in intensity with the increase of pullulan molecular weight as indicated by the red arrows (Figure 2A).

The exclusion rate of higher molar mass pullulans (i.e., 970 kDa & 1720 kDa) increased with TBAF concentration, but without affecting the major elution volume. The same phenomenon, i.e., the non-uniform effect on the higher- and lower-molecular weight macromolecules has been previously observed with pullulan standard solutions prepared in LiCl/DMAc, with an increased amount of LiCl in the standards solutions [33,34].

Despite these observations, the calibration curves obtained in these different conditions could be satisfactorily superimposed. As the one obtained in DMSO alone gave the most defined peaks and the higher correlation coefficient, it will be used as calibration curve for all the rest of the study.

### 2.2. Effect of TBAF Cencentration on AVICEL^®^ Chromatograms

**Figure 3:**
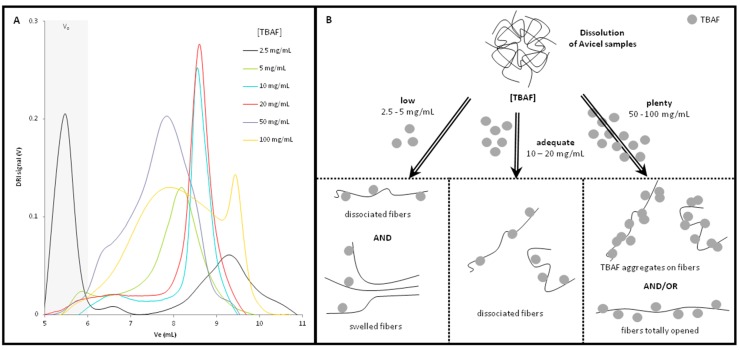
(**A**) Chromatograms of Avicel^®^ samples in different TBAF/DMSO solutions (DMSO; 60 °C; 0.5 mL/min) and (**B**) Representation of different TBAF concentration related phenomena affecting the elution volume, among to TBAF concentration.

It has been proven that, in organic salts solvent systems, the concentration of the solid salt in the organic solvent can affect the cellulose dissolution. Indeed, Strlič et al. [34] demonstrated that the increase of LiCl content in LiCl/DMAc solutions improves cellulose dissolution. Considering this, several Avicel^®^ samples in DMSO/TBAF have been prepared with different TBAF concentrations: from 2.5 mg/mL to 100 mg/mL. The obtained chromatograms are presented in Figure 3A. They demonstrated a strong influence of the TBAF concentration on the samples fractionation and hydrodynamic volumes.

For the lowest TBAF concentration (2.5 mg/mL) the elution profile presented two different peaks. The first one was eluted inside the exclusion volume and accounted for more than 50% of the signal. The second one, at high elution volume, was relatively large. Increasing the TBAF concentration (5 mg/L, 10 mg/L and 20 mg/L) a symmetric peak appeared between 7.5 mL and 9.5 mL, for 10 mg/L and 20 mg/mL this peak was particularly well defined and reproducible. Increasing further the TBAF concentration (50 mg/mL and 100 mg/mL), the chromatograms showed a widened peak indicating a large hydrodynamic volume dispersion inside this sample. For a TBAF concentration of 100 mg/L, even a second peak appeared for high elution volume.

Several hypotheses could explain these phenomena (Figure 3B). A too low TBAF concentration (below 5 mg/mL) does not seem to totally dissolve cellulosic chains, meaning one part of the sample may only be swollen. This swollen fraction, having a too high hydrodynamic volume to be retained in the column system, corresponds to the peak eluted in the exclusion volume as mentioned previously.

In ideal conditions, cellulose dissolution should be total, allowing a complete dissociation of polymeric chains, without influence of TBAF concentration on elution volumes. This seems to be the case for 10 mg/mL and 20 mg/mL, as both elution peaks were very similar.

Finally, for higher concentrations (50 mg/mL and 100 mg/mL), the excess of TBAF molecules involved a dispersion on hydrodynamic volume, that may be related either to their aggregation on dissociated macromolecules, or to total disruption of the intramolecular hydrogen bonds leading to the complete unfolding of cellulosic chains.

The molar mass obtained by conventional calibration with the calibration curve CC DT 0 confirms the phenomenon (Table 1).

In case of Avicel^®^ samples dissolved with 2.5, 5, 50 and 100 mg/mL, the obtained molar masses were higher than the ones given by the suppliers or measured by viscosimetry [26]. On the contrary, the values obtained for the samples 10 mg/mL and 20 mg/mL are consistent with the reference ones, confirming that with such TBAF concentrations, the solvent system completely dissolves Avicel^®^, without any apparent modification of its hydrodynamic volume.

Usually, the molar mass distribution determination using standard calibration curve requires working with the exact same dissolution conditions for both standards and samples. Accordingly, for the Avicel^®^ sample dissolved with a 10 mg/mL TBAF concentration, the average molar masses calculated using two different calibration curves (CC DT 0 and CC DT 1) were compared (Table 2).

Some caution is required when considering these values, because pullulan were used as standards as no cellulose standards were available on the market. As described by Berggren et al. [35], molar mass of cellulose samples determined by conventional calibration should be corrected by a factor determined by an absolute method based on light scattering. Nonetheless, the similarity of the obtained values supports the suitability of the CC DT 0 calibration curve for all samples dissolved in DMSO/TBAF system.

### 2.3. Chromatogram and Molar Mass Distribution of Cellulose Samples

**Figure 4:**
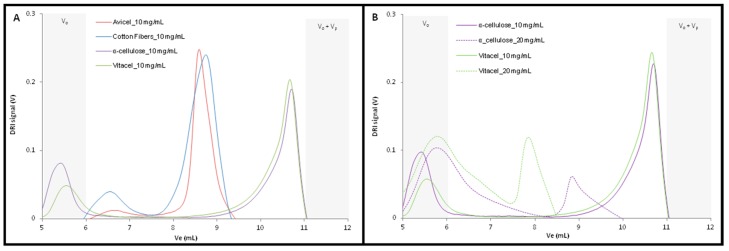
SEC chromatograms of cellulose samples dissolved with [TBAF] of 10 mg/mL and 20 mg/mL.

The four different cellulose samples were first dissolved into the solution of 10 mg/mL TBAF concentration. The chromatograms obtained presented two different behaviors depending on the celluloses nature (Figure 4A).

Avicel^®^ and cotton fibers, the lowest molar mass samples, had similar chromatograms displaying a well-defined peak between 8 mL and 9 mL, and a smaller one (especially for cotton fibers) between 6 mL and 7 mL, which is in accordance with their molar masses being close to each other.

The well-defined peaks indicated that the dissolution was completed and the fibers were totally dissociated during the process. For both samples, the peaks were in the range of the calibration curve and the excluded volume percentage was negligible, allowing the calculation of the average molecular weight (Table 3).

The obtained values were comparable to the viscosimetric ones and those obtained from the suppliers. Vitacel^®^ and α-cellulose, the highest molar mass samples, were also characterized by a similar DRI profile with two distinguished peaks identified on their respective chromatogram. But, the first peak was in the exclusion volume, whereas the second one, corresponding to very low hydrodynamic volume molecules, may be attributed to TBAF molecules complexed with DMSO, instead of cellulosic macromolecules (Figure 4A). Consequently, the average molecular weight were not calculated for these two samples.

A higher TBAF concentration (20 mg/mL) was then tested for α-cellulose and Vitacel^®^, in order to facilitate their dissolution and improve their fractionation. The obtained chromatograms (Figure 4B) presented again two distinct peaks. The first one was still eluted in the exclusion volume, but the second one was displaced to more reasonable elution volume (between 7.5 mL and 10 mL) corresponding probably to the totally dissolved cellulosic fraction. Unfortunately, the first peak accounted for more than 50% of the injected molecules (Table 2), preventing a correct calculation of the average molar masses, so the 20 mg/mL TBAF concentration did not seem sufficient to completely dissolve the high molecular weight cellulosic samples. For further investigation on analyses of high molecular weight cellulosic sample, it would hence be pertinent to test higher TBAF concentrations, keeping in mind that too high a concentration may lead to other artefacts during the fractionation.

## 3. Materials and Methods

### 3.1. Materials

Dimethyl sulfoxide (DMSO, 99.7%, anhydrous), tetrabutylammonium fluoride trihydrate (TBAF, 97%) and bis(ethylenediamine)copper(II) hydroxide solution (CED solution, 1 M in copper, molar ratio of ethylenediamine/copper of 2:1) were purchased from Sigma-Aldrich (Darmstadt, Germany). Four different samples of cellulose (one single batch each) have been studied with different average of DP (Table 1). Avicel^®^ (PH-101, Ph Eur, cellulose microcrystalline, batch n°BCBK2051V), α-cellulose (powder, batch n°BCBH3503V) and cotton fibers (cotton linters, medium fibers, batch n°MKBQ8042V) were purchased from Fluka (Sigma Aldrich, Darmstadt, Germany), and Vitacel^®^ (L600/30, Ph Eur, powdered cellulose, batch n°7120891215 X) from JRS Pharma (Rosenberg, Germany). Pullulan standards P 1720 kDa, P 970 kDa, P 636 kDa, P 318 kDa, P 184 kDa, P 100 kDa, P 45.5 kDa, P 20 kDa, P 9.2 kDa, P 5.9 kDa, P 1.1 Da and P 0.3 Da were purchased from Polymer Standards Service (PSS, Mainz, Germany).

### 3.2. Viscosimetric Measurements

Viscosimetric molar masses (Mv) of the different cellulosic samples, before and after dissolution in TBAF/DMSO solvent system, were determined indirectly by viscosimetric DP measurements of cellulose samples dissolved in the cupridiethylenediamine (CED). The samples were prepared and analyzed according to ASTM D-1795 [36].

### 3.3. Standard Procedure for Pullulan Dissolution in TBAF/DMSO

Pullulan standards were used to calibrate the column system. For each standard, the initial solution was prepared with 1.5 mg of pullulan samples placed under continuous magnetic stirring in 1.0 mL of DMSO during 30 min at ambient temperature.

To study the influence of the TBAF concentration on the molar mass distribution, three different pullulan standard ranges were prepared. Then, into each initial standard solution, 0.5 mL of TBAF/DMSO solution (containing 0 mg/mL, 15 mg/mL or 30 mg/mL of TBAF) were added. Finally, three SEC calibration curves with various TBAF concentrations (0, 5 and 10 mg/mL) were obtained (see Section 3.5).

### 3.4. Standard Procedure for Cellulose Dissolution in TBAF/DMSO

Two mg of cellulose samples with 10.0 g of DMSO were placed under continuous magnetic stirring during 2 h at 60 °C to swell the cellulosic fibers. Then, 10.0 g of TBAF/DMSO with 20 mg/mL and 40 mg/mL of TBAF were added. Finally, different solutions were obtained with 1 mg/mL of cellulose concentration and 10 mg/mL and 20 mg/mL of TBAF concentration, under the same stirring during 30 min. According to our previous study, the reaction temperature varied between 60 °C and 100 °C depending on the type of cellulose [26].

### 3.5. Size Exclusion Chromatography

The SEC system used consisted of a high-pressure isopump (G1310A, Agilent 1100 series, Agilent Technologies, Waldbronn, Germany), a stainless steel in-line filter with a 0.1 µm poly(tetrafluoroethylene) (PTFE) membrane (Millipore, Burlington, MA, USA), an automatic injector (G1313A, Agilent 1100 series, Agilent Technologies, Waldbronn, Germany), a column oven (G1316A, Agilent 1100 series, Agilent Technologies, Waldbronn, Germany), a SEC column packed with styrene-divinylbenzene (PS-DVB) copolymer gel (Mixed-B KD-806M, Shodex, München, Germany) preceded by a guard column (Mixed-B KD, Shodex) and a refractive index detector (RID-10A, Shimadzu, Kyoto, Japan). Data acquisition and processing were carried out using ASTRA software (Wyatt Technologies, Solvang, CA, USA).

SEC conditions were as follows: 1 mg/mL as sample concentration, 100 µL of injection volume, 0.5 mL/min flow rate and the column temperature set to 60 °C. The detector cell of RID was kept at 40 °C. The elution solvent was pure DMSO.

## 4. Conclusions

SEC analyses of various cellulosic samples were performed using TBAF/DMSO as solvent. Calibration curves from pullulan standards were successfully obtained, with different TBAF concentrations. The results underlined the impact of TBAF concentration on chromatographic profiles for both pullulan and cellulose samples.

For low molar mass cellulosic samples (Avicel^®^ and cotton fibers), this method allowed the determination of the average molecular weight by conventional calibration. For higher molar mass cellulosic samples, the tested TBAF concentration range was not optimal which prevented the correct calculation of their average molecular weight. The saturation of the column by TBAF molecules did not allow to use TBAF/DMSO solutions as elution solvent. Improvements are still needed to apply a correction factor to the calibration curves and/or to consider the use of absolute method based on light scattering (MALS detector).

## Figures and Tables

**Table 1:** Influence of TBAF concentrations on MM distribution analyses of Avicel^®^ samples.

Sample	(TBAF) (mg/mL)	%Excl. ^a^	Dispersity	M*n* (kDa)	M*w*	M*v* (kDa)		Supplier MM (kDa)	
						*Untreated*	Dissolved *	*Inferior*	*Superior*
Avicel^®^	100	2.4	2.7	82.1	221.3	36.6	47.8	24.3	56.7
			1.1	12.6	14.2				
	50	3.5	2.1	110.0	233.4				
	20	0.9	1.1	43.6	48.4				
	10	0.8	1.1	43.8	46.9				
	5	1.3	1.3	68.2	87.0				
	2.5	64.5	nd	nd	nd				

^a^ %Excl. is the no-retention percent of the sample; * After dissolution in DMSO/TBAF as described in our previous work [26]; nd: not determined.

**Table 2:** Determination of molar masses among two different calibration curves of Avicel^®^ samples dissolved with 10 mg/mL of TBAF concentration in solution.

	%Excl. ^a^	Dispersity	M*n* (kDa)	M*w* (kDa)
C DT 0	0.8	1.1	43.8	46.9
CC DT 1	0.9	1.1	45.7	49.8

^a^ %Excl. is the no-retention percent of the sample.

**Table 3:** Molar masses of cellulose samples dissolved with 10 mg/mL and 20 mg/mL TBAF concentration.

Sample	[TBAF] (mg/mL)	%Excl. ^a^	Dispersity	M*n* (kDa)	M*w* (kDa)	M*v* (kDa)		Supplier MM (kDa)	
						*Untreated*	*Dissolved* *	*Inferior*	*Superior*
Avicel	10	0.8	1.1	43.8	46.9	36.6	47.8	24.3	56.7
Cotton Fibers	10	1.2	1.2	38.4	47.6	43.9	44.4	30.8	48.6
α-cellulose	20	62.3	nd	nd	nd	196.3	190.2	113.1	194.2
Vitacel	20	52.6	nd	nd	nd	174.1	195.2	71.3	365.2

^a^ %Excl. is the no-retention percent of the sample; * After dissolution in DMSO/TBAF as described in our previous work [26]; nd: not determined.
